# Coherence of Visual-Evoked Gamma Oscillations Is Disrupted by Propofol but Preserved Under Equipotent Doses of Isoflurane

**DOI:** 10.3389/fnsys.2019.00019

**Published:** 2019-05-08

**Authors:** Adeeti Aggarwal, Connor Brennan, Brenna Shortal, Diego Contreras, Max B. Kelz, Alex Proekt

**Affiliations:** ^1^Department of Neuroscience, Perelman School of Medicine, University of Pennsylvania, Philadelphia, PA, United States; ^2^Department of Anesthesiology and Critical Care, Perelman School of Medicine, University of Pennsylvania, Philadelphia, PA, United States

**Keywords:** isoflurane, propofol, gamma, VEP, visual evoked potential, anesthesia, burst suppression, brain state

## Abstract

Previous research demonstrates that the underlying state of the brain influences how sensory stimuli are processed. Canonically, the state of the brain has been defined by quantifying the spectral characteristics of spontaneous fluctuations in local field potentials (LFP). Here, we utilized isoflurane and propofol anesthesia to parametrically alter the spectral state of the murine brain. With either drug, we produce slow wave activity, with low anesthetic doses, or burst suppression, with higher doses. We find that while spontaneous LFP oscillations were similar, the average visual-evoked potential (VEP) was always smaller in amplitude and shorter in duration under propofol than under comparable doses of isoflurane. This diminished average VEP results from increased trial-to-trial variability in VEPs under propofol. One feature of single trial VEPs that was consistent in all animals was visual-evoked gamma band oscillation (20–60 Hz). This gamma band oscillation was coherent between trials in the early phase (<250 ms) of the visual evoked potential under isoflurane. Inter trial phase coherence (ITPC) of gamma oscillations was dramatically attenuated in the same propofol anesthetized mice despite similar spontaneous oscillations in the LFP. This suggests that while both anesthetics lead to loss of consciousness (LOC), elicit slow oscillations and burst suppression, only the isoflurane permits phase resetting of gamma oscillations by visual stimuli. These results demonstrate that accurate characterization of a brain state must include both spontaneous as well as stimulus-induced perturbations of brain activity.

## Introduction

Anesthesia is a staple in modern healthcare due to its ability to provide a reversible state of unconsciousness, which is essential for painless surgery and for sedation in intensive care units (ICUs). Anesthetics have also proved indispensable for basic neuroscience. Indeed, much of our knowledge concerning sensory processing is derived from experiments performed in anesthetized animals (Mountcastle, 1957; Hubel and Wiesel, 1962; Destexhe et al., 1999). Despite their widespread use, the mechanisms by which anesthetics produce a reversible loss of consciousness (LOC) remain unknown. One practical implication of this knowledge gap is that clinical monitoring of the anesthetized state is unable to guarantee that all patients are, in fact, unconscious during surgery. While depth of anesthesia monitors do ensure that majority of anesthetized patients are unconscious, 4–10% of patients under general anesthesia exhibit a covert return of consciousness as evidenced by their ability to follow simple verbal commands (Russell, 1989; Schneider Gerhard et al., 2005; Mashour et al., 2011; Sanders et al., 2012). Current EEG-based “depth of anesthesia” devices do not reliably detect these episodes of awareness (Mashour, 2006; Sanders, 2016). While patients with covert awareness are less likely to form memories (Mashour et al., 2011; Mashour et al., 2012; Sanders et al., 2012), up to 70% of those that do, develop long-lasting psychiatric consequences such as post-traumatic stress disorder (PTSD) (Leslie et al., 2010).

General anesthetic agents are structurally heterogeneous and exhibit promiscuous binding to a wide variety of molecular targets (Eckenhoff and Eckenhoff, 1998; Lydic and Baghdoyan Helen, 2005; Franks, 2008). It is highly unlikely that each anesthetic drug disrupts consciousness using the same molecular effectors. Nevertheless, mechanistically distinct anesthetics are known to generate similar patterns of brain activity. The most prevalent pattern of brain activity observed in the anesthetized brain are the canonical slow oscillations first demonstrated in human EEG in the 1930’s (Gibbs et al., 1937). For example, propofol, a positive allosteric modulator at GABA_A_ receptors (Jurd et al., 2003), induces low frequency large amplitude EEG oscillations and sleep-like spindles (Ching et al., 2010; Purdon et al., 2013). Likewise, the inhaled anesthetic, isoflurane, also produces slow wave activity with distinct UP-states and DOWN-states in the EEG (Ferron et al., 2009). While isoflurane also acts on the GABA_A_ receptor (Hall et al., 1994), its actions on the GABA_A_ receptor are distinct from those of propofol (Krasowski et al., 1998). Furthermore, actions of isoflurane on the GABA_A_ receptor appear to be less critical for its ability to induce anesthesia than those of propofol (Sonner et al., 2007). Finally, both propofol and isoflurane interact with a number of other receptors in the nervous system (Eckenhoff, 2002; Weiser et al., 2015).

It is thought that the slow EEG oscillations observed with a variety, but notably not all anesthetics (Maksimow et al., 2006; Akeju et al., 2016), are a consequence of a switch in the activity patterns of thalamic neurons. These neurons shift from tonic firing, which denote awake desynchronized states, to bursting firing pattern, which synchronizes cortical activity. Thalamic bursting activity is thought to prevent reliable transmission of sensory stimuli from the thalamus to the cortex. This hypothesis suggests that, regardless of the molecular mechanism of action, slow oscillations induced by mechanistically distinct anesthetics should lead to similar disruptions of sensory-evoked responses in the cortex.

There is recent evidence, however, to challenge this hypothesis. Arena et al demonstrate that the amplitude of visual-evoked potentials (VEPs) is attenuated by propofol, but enhanced by increasing concentrations of sevoflurane in rats (Arena et al., 2017). Here, we build upon these observations and characterize the differences in visual-evoked responses in mice under isoflurane and propofol. We find that although there are similarities in the spontaneous activity elicited by hypnotic doses of isoflurane and propofol, visual-evoked responses to simple visual stimuli are quite different in the primary visual cortex (V1). In the time domain, we find that responses evoked by identical visual stimuli vary dramatically between trials under both anesthetics. However, analysis in the frequency-domain reveals a consistent visual-evoked gamma oscillation (20–60 Hz) present in all mice. This gamma oscillation is coherent across trials in the early phase (<250 ms) of the VEP when mice are under isoflurane anesthesia. Despite similar drug-induced brain states, visual-evoked gamma coherence between trials is greatly attenuated when the same mice are anesthetized with steady-state, target controlled infusions of propofol. This suggests that while both anesthetics disrupt consciousness and elicit slow oscillations, only the isoflurane-induced state of unconsciousness permits phase resetting of gamma oscillations by visual stimuli.

## Results

To elucidate the effect of the anesthetic state on visual-evoked brain activity, we performed *in vivo* electrophysiological recordings in mice head-fixed with ear bars (*n* = 7) presented with simple visual stimuli using equipotent doses of two different anesthetic drugs. Each visual stimulus consisted of a short, 10 ms, flash of a green LED light that covered 100% of the right visual field. Spontaneous and evoked local field potentials (LFP) were collected using an electrocorticography (ECoG) electrode placed on top of the dura over the left hemisphere, including primary visual cortex.

### Survey of Spectrally Defined Brain States Under Isoflurane and Propofol

In order to modulate the spontaneous cortical activity, we delivered two different anesthetics: isoflurane and propofol. We were able to maintain steady state concentration of isoflurane via a nose cone delivery due to its relatively fast pharmacokinetics. Propofol was administered intravenously (IV) through a jugular venous catheter with a target controlled infusion (TCI) to ensure that the propofol brain concentration remained constant (Shortal et al., 2018).

**FIGURE 1:**
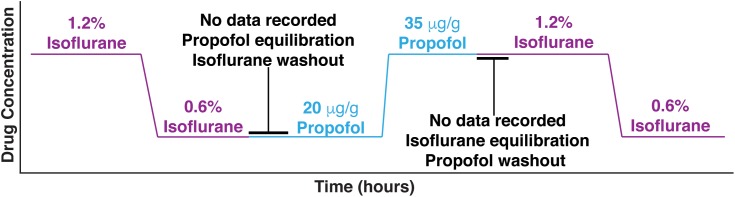
Experimental design. Mice were first given two doses of isoflurane (high – 1.2%, and low – 0.6%), then given two doses of propofol (low – 20 μg/g brain and high – 35 μg/g brain). Between isoflurane and propofol recordings, the brain was allowed 45 min to wash out isoflurane and establish equilibrium with propofol. Four out of 7 mice were re-exposed to the high and low isoflurane doses after propofol was washed out for 1 h.

**FIGURE 2:**
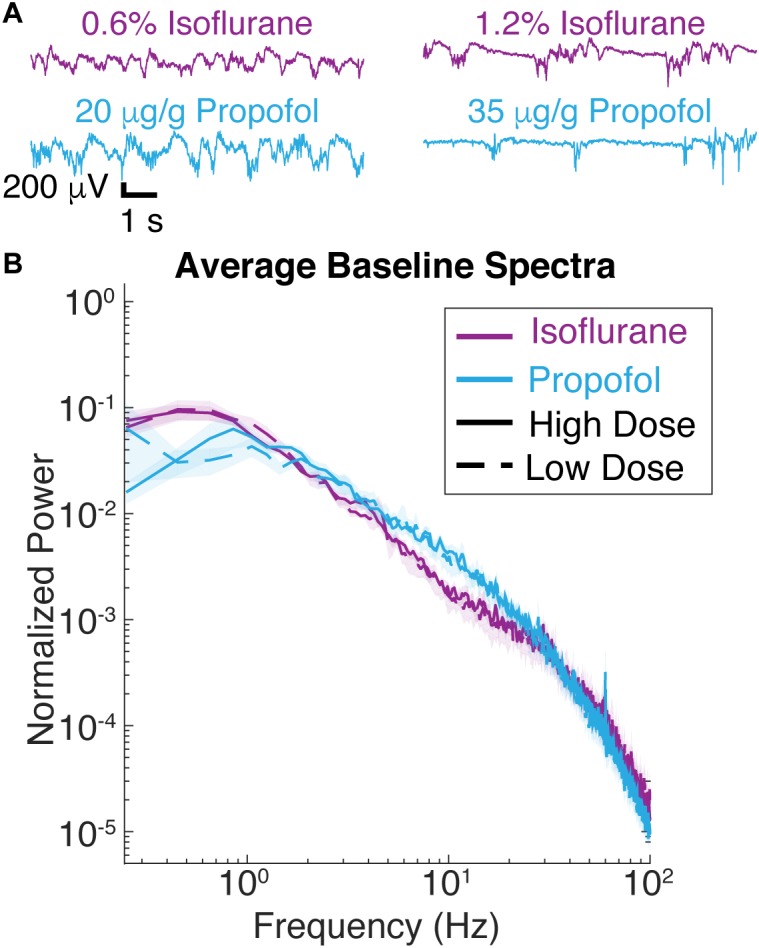
Spontaneous LFP of mice under isoflurane and propofol have similar spectral characteristics. **(A)** Ten seconds of unstimulated local field potential (LFP) recorded in V1 under high dose isoflurane (1.2%), low dose isoflurane (0.6%), high dose propofol (35 μg/g brain), low dose propofol (20 μg/g brain). **(B)** Power spectra of 1 min of unstimulated LFP from V1 were computed for all seven animals. Blue curves are from animals under propofol, while purple traces are from animals under isoflurane. Solid lines denote high drug concentrations while dashed denote low drug concentrations. Shading represents the 95% confidence intervals for each condition.

Three out of seven mice were first given two doses of isoflurane (high – 1.2%, and low – 0.6%), then given two doses of propofol (low – 20 μg/g brain and high – 35 μg/g brain). The remaining four mice had the aforementioned exposure and were subsequently re-exposed to the same two doses of isoflurane 1 h after propofol was washed out. Re-exposure served as a control for the potential brain desiccation, which might occur during long recording sessions. Re-exposure experiments also established the consistency of specimen preparation (Figure 1). At every anesthetic dose, 1 min of spontaneous activity was collected before visual stimuli were presented. Ten seconds of spontaneous data is shown in Figure 2A, illustrating that burst suppression occurs with high doses of both propofol [median suppression ratio (*SR*) = 6.65%, interquartile range (IQR) = 25.09%] and isoflurane (median SR = 9.55%, IQR = 14.74%); and that large amplitude slow waves arise with low doses of propofol (median *SR* = 0.80%, IQR = 4.23%) and isoflurane (median *SR* = 1.15%, IQR = 4.23%).

Consistent with these observations, spectra of the ECoG signals under isoflurane and propofol overlap over frequencies ranging from 1 to 4 Hz (Figure 2B). While with respect to slow wave activity ECoG spectra under propofol and isoflurane anesthesia were highly similar, there was slightly more power at frequencies between 6 and 10 Hz under propofol. There was also an increase 0.3–1 Hz under isoflurane compared to propofol (*df* = 3, *n* = 7, *p* = 0.011, Kruskal–Wallis, pooled isoflurane vs. pooled propofol, *n* = 7, *p* = 0.005, Mann–Whitney *U*-test with *post hoc* Bonferroni Correction). Thus, by administering the same animal with these two chemically distinct anesthetics, we can determine how similar slow oscillations induced with two distinct anesthetics affect the characteristics of visual-evoked responses.

### Isoflurane and Propofol Have Dramatically Different Average Visual-Evoked Responses

**FIGURE 3:**
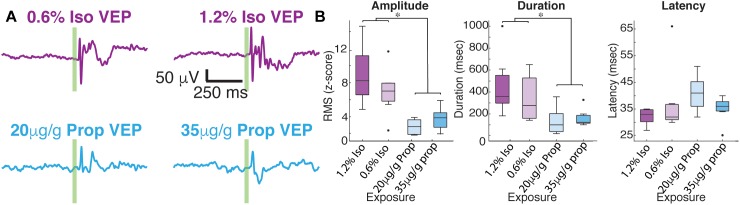
Average visual evoked responses under isoflurane and propofol are dramatically different within the same animal. **(A)** Average of 100 flash trials under each does of isoflurane (top) and propofol (bottom). The flash is denoted by the green vertical line. **(B)** Quantification of average VEP amplitude (*n* = 7, *p*-value amplitude < 0.001, Mann–Whitney *U*-test with *post hoc* Bonferroni Correction), duration of VEP (*n* = 7, *p*-value duration = 0.003, Mann–Whitney *U*-test with *post hoc* Bonferroni Correction), and latency of onset (*df* = 3, *n* = 7, *p*-value = 0.044, Kruskal–Wallis). asterisks (^∗^) denote *p* < 0.01.

After 1 min of baseline recording, 100 visual-evoked responses were elicited using a green LED. Averaged VEPs under each anesthetic condition are shown in Figure 3. The shape of average VEP has historically been described by its latency to onset, amplitude, and response duration. We defined the latency to onset of the VEP as statistical deviations from the pre-stimulus data (Materials and Methods). Similarly, we define the duration of VEPs by the number of timepoints for the post-stimulus data to recapitulate the pre-stimulus statistics (Materials and Methods). We defined the amplitude of the VEP as the root mean square (RMS) of the first 350 ms of post-stimulus data. No changes in the overall amplitude of the VEP were found for different concentrations of the same anesthetic (low dose isoflurane vs. high dose isoflurane: *n* = 7, *p* = 0.999, low dose propofol vs. high dose propofol, *n* = 7, *p* = 0.902, Mann–Whitney *U*-test). When we normalize the evoked RMS to the RMS calculated from baseline, we still observe that the overall amplitude of the VEP was similar under the two concentrations of isoflurane (low dose isoflurane vs. high dose isoflurane: *n* = 7, *p* = 0.383). Low dose propofol was associated with small but statistically significant decrease in the VEPs relative to high dose propofol (*n* = 7, *p* < 0.001, Mann–Whitney *U*-test). Furthermore, we were not able to detect differences in duration of VEP at different anesthetic concentrations (low dose isoflurane vs. high dose isoflurane, *n* = 7, *p* = 0.383; low dose propofol vs. high dose propofol, *n* = 7, *p* = 0.209, Mann–Whitney *U*-test, with *post hoc* Bonferroni Correction). In contrast, differences in VEP characteristics were strongly dependent on the identity of the anesthetic agent. The high and low doses of drugs were combined since there were no dose dependent differences. VEPs under propofol were smaller in amplitude (*n* = 7, *p*-value amplitude < 0.001, Mann–Whitney *U*-test with *post hoc* Bonferroni Correction) and shorter in duration (*n* = 7, *p*-value duration = 0.003, Mann–Whitney *U*-test with *post hoc* Bonferroni Correction) than under isoflurane. Kruskal–Wallis *U*-test of the latencies for all four drug conditions was borderline statistically significant (*df* = 3, *n* = 7, *p-*value = 0.044). None of the *post hoc* Mann–Whitney *U*-tests for pairwise comparisons between drug conditions reached statistical significance.

### Large Trial-to-Trial Variability Under Both Anesthetics

**FIGURE 4:**
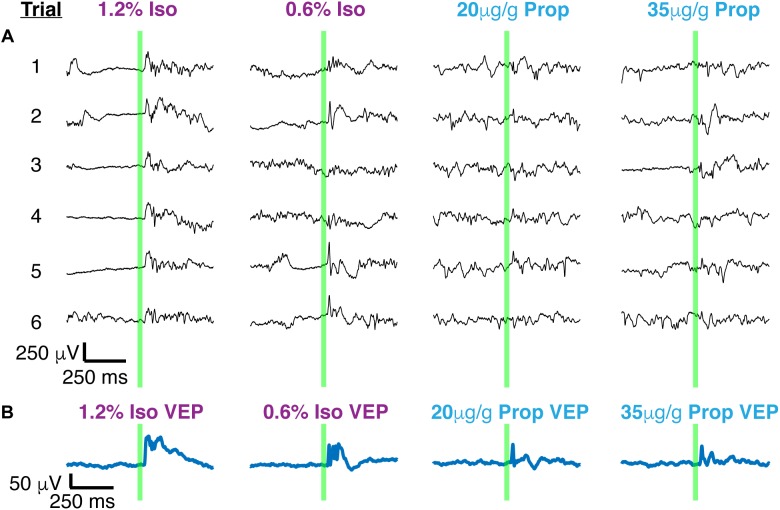
Single trials of visual evoked potentials under isoflurane and propofol both have high trial by trial variability. **(A)** Six out of 100 randomly chosen individual flash evoked potentials (thin black traces) under high and low doses of each anesthetic: isoflurane, propofol. **(B)** The average VEP over 100 trials for each anesthetic concentration. The flash is denoted by the green vertical line in both panels.

Two distinct scenarios can potentially give rise to the observed differences in the amplitude and duration of the average VEPs: (1) VEPs could be larger in individual trials under isoflurane than under propofol, (2) VEPs could be more consistent among trials under isoflurane. To differentiate between these possibilities, we first surveyed the single trial visual-evoked responses. We found that single trials exhibit large trial-by-trial variability and are strongly dominated by ongoing spontaneous brain activity under both isoflurane and under propofol (Figure 4A). We were unable to unequivocally determine the latency of onset or duration of the VEP on a single trial basis since the post-stimulus signal did not deviate significantly from pre-stimulus ECoG. Moreover, we could not find a difference in the amplitude of the single trial responses under isoflurane and propofol (χ^2^ = 1.05, *df* = 3, *n* = 7, *p* = 0.197, Kruskal–Wallis). Despite this inter-trial variability, averaging across all 100 trials reveals a clear, VEP. The shape of the average evoked potential, however, rarely resembles any individual trial (Figure 4B). Moreover, individual visual evoked trials do not have the same waveform in the time domain. We measured the average pairwise correlation between single trials as an indicator of reliability under each dose of each anesthetic (Tiesinga et al., 2003; Kumbhani et al., 2007). In aggregate, in the time domain, visual evoked single trials are weakly correlated with each other under both anesthetics. Yet, there is a significant increase in the reliability under isoflurane (pooled mean reliability under isoflurane = 0.205) than under propofol (pooled mean reliability under isoflurane = 0.063) (*n* = 7, *p*-value pooled reliability < 0.001, Mann–Whitney *U*-test with *post hoc* Bonferroni Correction). This suggests, that the observed differences in the average VEPs under isoflurane and propofol are likely due to differences in the inter-trial consistency of responses rather than to differences in the shape of the VEP on individual trials.

### Visual-Evoked Gamma Power Under Isoflurane and Propofol

**FIGURE 5:**
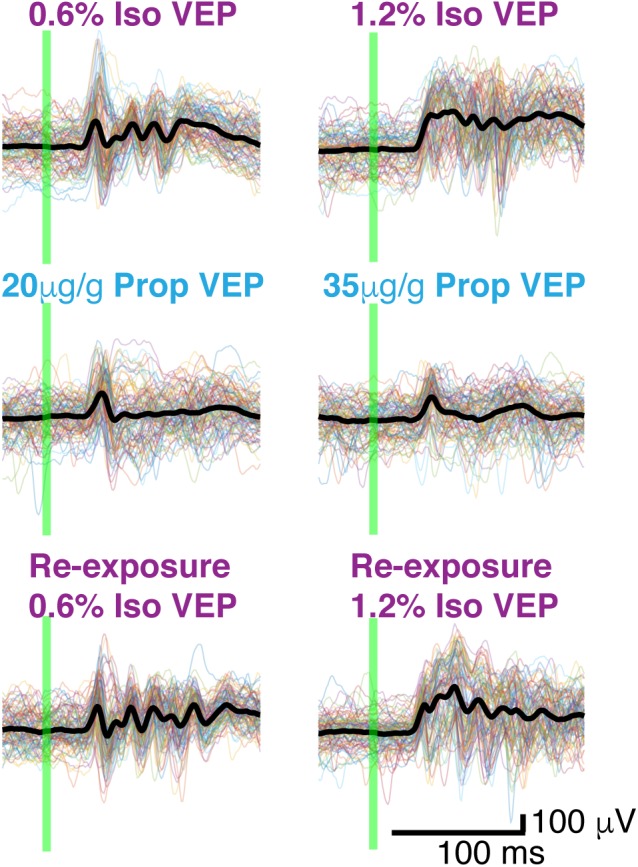
Butterfly plots. Thin colored traces under high and low doses of each anesthetic: isoflurane, propofol, and isoflurane re-exposure in the same animal. Thick black lines represent the average VEP under each dose of each anesthetic. The flash is denoted by the green vertical line.

**FIGURE 6:**
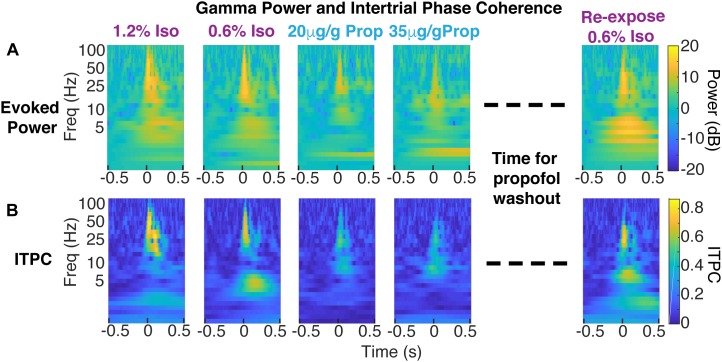
Decrease in coherent evoked gamma power in propofol compared to isoflurane within the same animal. **(A)** Color plot of average evoked power (first isoflurane exposure in the left panels, propofol exposures in the middle panels, and re-exposure to isoflurane acquired 1 h after propofol wash out in the right panels). **(B)** Color plot of ITPC.

While VEPs vary dramatically between trials in many respects, one aspect of the VEP –an oscillation around 40 Hz – was highly consistent between trials and was present in all mice (Figure 5). This gamma oscillation can be clearly visualized in the frequency domain (Figure 6). To extract the frequency, power and phase characteristics of oscillations present in visual-evoked responses, we convolved single trials with a series of Morlet wavelets. Spectra averaged across trials were then normalized to the pre-stimulus interval (Figure 6A). On average, over the first 250 ms, higher gamma power (20–60 Hz), was evoked by the visual stimulus under isoflurane than under propofol (timepoints = 900, *p* < 0.000001, Mann–Whitney *U*-test).

To determine the variability of the phase of the visual-evoked gamma oscillation, we computed inter-trial phase coherence (ITPC) at each point in the time-frequency plane. Consistent with the observations in the time domain (Figure 5), ITPC was significantly increased in the gamma range following the visual stimulus. This increase in the ITPC was most prominent between 50 and 250 ms after stimulus between 20 and 60 Hz (Figure 6B). Moreover, the increase in ITPC was larger during anesthesia with isoflurane than propofol. Propofol’s reduced ITPC recovered following washout of propofol and re-exposure to isoflurane. Note, that coherence is normalized to signal power. Thus, differences in the ITPC cannot be attributed to higher power of gamma oscillations under isoflurane.

**FIGURE 7:**
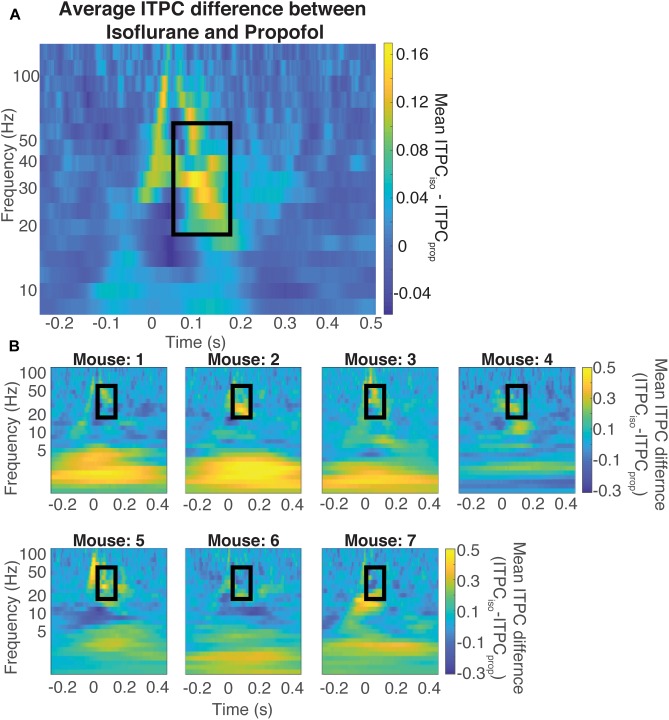
Difference in coherence is in the evoked gamma band. Average difference between the ITPC under both doses of isoflurane and propofol **(A)** Yellow colors represent higher ITPC under isoflurane while dark blue colors represent higher ITPC under propofol. The maximum difference in evoked coherence occurs within the black rectangle, at 80 ms after stimulus onset and is centered at 36 Hz. The Quantification of the ITPC in the gamma range (20–60 Hz) within the black rectangle yields a significant difference between the gamma coherence of visual evoked responses (timepoints = 900, *p* < 0.000001, Mann–Whitney *U*-test). Individual difference between the ITPC under both doses of isoflurane and propofol **(B)**. Yellow colors represent higher ITPC under isoflurane while dark blue colors represent higher ITPC under propofol. The Quantification of the ITPC in the gamma range (20–60 Hz) within the black rectangle yields a significant difference between the gamma coherence of visual evoked responses.

Figure 7 shows the difference between ITPC evoked under isoflurane and propofol. Here, both high and low concentrations for each individual anesthetic were combined, the anesthetic agent effects are larger than the concentration-dependent effects (Table 1). Yellow colors represent higher ITPC under isoflurane while dark blue colors represent higher ITPC under propofol. The maximum difference in evoked coherence occurred 80–130 ms after stimulus onset and was centered at 36 Hz. Indeed, we found a significant increase in the ITPC under isoflurane compared to propofol (timepoints = 900, *p* < 0.000001, Mann–Whitney *U*-test). Significant decrease in ITPC in the gamma range were present in each mouse (Table 2). In 6 out of 7 mice, the visual evoked gamma coherence is statistically greater under isoflurane than under propofol anesthesia with *post hoc* Bonferroni correction. In contrast to the consistent increase in gamma coherence following the visual stimulus, the increase in coherence at lower frequencies (1–5 Hz, centered at 3 Hz) was not consistent among animals. Moreover, none of the average VEPs exhibited a clear oscillation in the 1–5 Hz range that lasted for one or more cycles.

**Table 1:** Difference in evoked gamma coherence across mice (all *p*-values are Bonferroni corrected unless otherwise specified).

Comparison	Mann–Whitney *U*-test *p*-value for ITPC difference
Pooled isoflurane – pooled propofol	<0.000001
Low dose isoflurane – high does isoflurane	Uncorrected *p*-value = 0.0611
Low dose propofol – high does propofol	<0.000001
Low dose isoflurane – low does propofol	<0.000001
High dose isoflurane – high does propofol	<0.000001


**Table 2:** Individual mouse differences in evoked gamma coherence from isoflurane – propofol (all *p*-values are Bonferroni corrected unless otherwise specified).

Mouse	Mann–Whitney *U*-test *p*-value for ITPC difference
1	0.336
2	<0.000001
3	<0.000001
4	<0.000001
5	<0.000001
6	<0.000001
7	<0.000001


## Discussion

Maintenance of a stable perceptual world is a fundamental requirement of consciousness. In order to create such stable representation of the sensory stimuli, sensory information must be faithfully relayed and integrated with ongoing spontaneous brain activity. The mechanisms through which general anesthetics disrupt perception remain a mystery. Furthermore, it is unknown whether mechanistically distinct classes of anesthetics disrupt sensory processing in a similar manner. Here, we show that although two chemically distinct anesthetics, isoflurane and propofol, produce similar spontaneous ECoG activity, visual-evoked responses recorded in primary visual cortex obtained during each anesthetic state are quite different. When mice are anesthetized with isoflurane, there is a consistent visual-evoked gamma band oscillation (20–60Hz), which is synchronous across trials. However, when the same mice are anesthetized with propofol, visual-evoked gamma coherence between trials is greatly attenuated. This decrease in consistency of visual responses to identical stimuli likely contributes to the decrease in the size and duration of the visual-evoked responses under propofol. Curiously, both anesthetics elicit similar oscillations in the spontaneous LFP. For instance, under high concentrations of both propofol and isoflurane, the LFP was characterized by burst suppression. Yet, the consistency of elicited responses varied dramatically depending on whether propofol or isoflurane was used to elicit burst suppression. These observations complicate analysis of “brain state” under anesthesia on the basis of spontaneous oscillations in the LFP.

### Sensory Neurophysiology Research Under Anesthesia

For decades, much of sensory neurophysiology research has been performed in anesthetized preparations. There is increasing evidence that anesthetized and awake sensory responses differ greatly (Imas et al., 2005; Reinhold et al., 2015; Storchi et al., 2017). For example, visual cortical responses quickly adapt to a train of high frequency visual stimuli when mice are under isoflurane compared to the awake state (Reinhold et al., 2015). This adaptation is thought to occur because under isoflurane, there is synaptic depression at the level of the lateral geniculate thalamic cells (Reinhold et al., 2015). Furthermore, responses in V1 depend strongly on the behavioral state such as resting vs. running (Niell and Stryker, 2010). It is less obvious, however, that responses to simple visual stimuli in V1 should depend strongly on the anesthetic agent. The fact that responses to a simple flash in V1 depend strongly upon whether propofol or isoflurane was used to maintain anesthesia is especially surprising because the spontaneous fluctuations in brain activity produced by these anesthetics are very similar.

It is often difficult to determine how animals were anesthetized in existing literature. Materials and Methods sections sometimes note the fluctuations in the spontaneous EEG as a proxy for defining the brain state at the time of recording. However, given the results presented here, along with findings by others (Arena et al., 2017), the type of anesthetic can dramatically alter sensory responses even when the spontaneous oscillations in the ECoG signals are similar. Thus, the traditional characterization of the oscillations of spontaneous brain activity does not appear to unequivocally specify the characteristics of responses evoked by the visual stimulus.

### Possible Mechanisms of Gamma Coherence Breakdown Under Propofol

Variability of evoked responses to identical sensory stimuli limits the ability of these response to reliably convey information about stimulus attributes. Currently, it is not understood which parameters of the visual evoked response encode sensory information. We find that the animal-to-animal and trial-to-trial variability of the visual evoked response decreases in the phase of the visual-evoked gamma oscillations. This makes the phase of the gamma oscillations an appealing candidate for encoding the visual stimulus. Gamma activity can be elicited by one of two prevailing mechanisms. The first arises from strongly activating interneuron-interneuron networks (I-I) (Wang and Buzsáki, 1996; Kopell et al., 2000; Ermentrout and Kleinfeld, 2001; Vinck et al., 2015). The second comes from reciprocally activating interneurons and pyramidal neurons (E-I) (Wilson and Cowan, 1972; Traub et al., 1996; Whittington et al., 2000; Cardin, 2016; Friston and Buzsáki, 2016; Sohal, 2016; Traub et al., 2016). Critical for both of these mechanisms is the shape of the IPSPs produced by fast spiking GABAergic interneurons. Propofol allosterically potentiates GABA signaling through the GABA_A_ receptor (Hales and Lambert, 1991; Krasowski et al., 1998; Yip et al., 2013; Weiser et al., 2015). Therefore, under propofol, the duration of the IPSPS may be prolonged (Bai et al., 1999). This may be why we see a decrease in total gamma power under propofol, and in some animals (for example, shown in Figure 6), we see a shift to lower evoked gamma power. However, one caveat to this hypothesis is that isoflurane also is a positive allosteric modulator at synaptic and extrasynaptic GABA_A_ receptors (Krasowski et al., 1998; Wang, 2009; Garcia et al., 2010). Isoflurane suppresses GABAergic IPSPs at lower concentrations (Jones and Harrison, 1993; Pearce, 1996; Banks and Pearce, 1999) but, in a hippocampal slice preparation concentrations similar to that used in this study increased the amplitude and duration of GABAergic IPSPs (Miu and Puil, 1989). Yet, other studies in amygdalar slices suggest that isoflurane prolongs GABA_A_ mediated currents without effectively increasing their amplitude (Ranft et al., 2004). Both isoflurane and propofol are highly promiscuous drugs that have significant interactions with a host of membrane proteins (Eckenhoff, 2002; Weiser et al., 2015; Tang and Eckenhoff, 2018). Thus, in the absence of detailed biophysical model, which would include actions of both propofol and isoflurane at the plurality of their molecular targets, it is difficult to attribute the differences in the visually induced gamma oscillations to specific differences in the molecular mechanisms of action of either anesthetic. Perhaps, most surprising is the difference in the visual-evoked responses under deep anesthesia characterized by burst suppression. *In vivo* extracellular and intracellular recordings in cats suggest that burst suppression induced with either propofol (Kroeger and Amzica, 2007) or with isoflurane (Kroeger and Amzica, 2007; Amzica, 2009; Ferron et al., 2009) is associated with hyper-excitability of the cortex. Our observations concur with that of Amzica and colleagues – visual stimuli presented under both isoflurane-induced and propofol-induced burst suppression during the suppression period of the ECoG could on occasion trigger bursts (Kroeger and Amzica, 2007; Amzica, 2009; Ferron et al., 2009). Yet, our results suggest that burst suppression induced with isoflurane allows visual stimuli to entrain the phase of the gamma oscillations while burst suppression induced with propofol does not.

Exactly how the laminar structure of the primary visual cortex generates spontaneous and induced gamma rhythms in V1 is also unknown. There is some evidence that the granular layer of V1 is more resistant spectral composition changes under a mixture of isoflurane and xylazine as compared to higher order brain regions in ferrets (Sellers et al., 2013). Moreover, mice under isoflurane/xylazine, tend to have spontaneous gamma waves that begin in all layers simultaneously, however, visual evoked gamma oscillations begin in granular and supra-granular layers (Welle and Contreras, 2015). This may be because isoflurane increases the number of excitatory and inhibitory cells that are recruited to participate in synchronous responses, as seen in Noda and Takahashi (2015). This would also correspond well to findings seen in ferrets which show that visual evoked multiunit activity have a longer duration when animals are anesthetized with isoflurane/xylazine compared to in the awake state. Interestingly, just because gamma oscillations are seen in the superficial layers does not necessarily mean that there is in fact gamma oscillations in the deeper layers, or that there is strong synchrony between spiking activity and phase of the gamma oscillation (Sellers et al., 2015; Welle and Contreras, 2015).

Visual-evoked gamma power and frequency has also been shown to increase with arousal and locomotion in mice (Niell and Stryker, 2010; Polack et al., 2013; Fu et al., 2014). While we can rule out locomotor effects given our anesthetized preps, the activation of arousal circuitry may be different under these two anesthetics. Indeed, when mice have larger pupillary diameters, indicating increased arousal (Aston-Jones and Cohen, 2005; Gilzenrat et al., 2010; Eldar et al., 2013), visual evoked responses have large signal to noise ratio, and exhibit two peaks of visual evoked gamma power centered around 75 and 30 Hz (Vinck et al., 2015). These effects may be caused by neuromodulation from sleep and arousal systems. For example, the mix of cholinergic tone and noradrenergic input has been shown to maintain high signal to noise seen in V1 neurons during arousal and locomotion in mice (Polack et al., 2013). Moreover, cholinergic projections from the basal forebrain have been shown to increase visual evoked gamma oscillations in mice (Pinto et al., 2013; Lee et al., 2014). Our results thus may indicate that propofol may depress arousal circuitry involving cholinergic and noradrenergic input more than isoflurane. Thus, understanding how anesthetics alter sensory responses may help us formulate hypotheses about the mechanisms by which anesthetics affect the sleep and arousal system to differentially produce unconsciousness.

### Functional Implications

The precise functional implications of visual-evoked gamma oscillations are currently unknown. Some evidence suggests that the sensory evoked gamma oscillations modulate the firing of sensory neurons and increase the efficiency of sensory encoding (Womelsdorf et al., 2006; Cardin et al., 2009; Womelsdorf et al., 2012). This may occur through decreasing noise by increasing inhibitory drive, or increasing signal by entraining sensory evoked firing to a specific phase of the gamma oscillation (Fries et al., 2001; Cardin et al., 2009; Sohal, 2016). It is important to note that we not only observe visual evoked gamma oscillations in the single trial data, and increase in gamma power, but also that this increase in gamma power is consistent in phase from trial to trial under isoflurane. This implies that the neural processes leading to visual evoked responses occur in a stereotyped fashion under isoflurane compared to propofol anesthesia. A possible mechanism leading to such a phase resetting effect would be strong synchrony in visual cortex neuronal firing under isoflurane compared to propofol. Indeed, in ferrets given isoflurane and xylazine, visual stimuli increase ITPC in V1, however, when the same animals are awake there is an increase in ITPC both in V1 and in the PFC, thereby indicating that such phase coherence may be important for functional connectivity between different regions of the brain (Sellers et al., 2015). However, it is not yet clear if this increase in phase synchrony across the brain will necessarily be able to provide more information for encoding stimulus attributes. For example, there is an increase in auditory evoked gamma coherence between different areas of rat auditory cortex and belt in rats under isoflurane anesthesia as compared to awake rats (Noda and Takahashi, 2015).

### Limitations

While visual-evoked gamma oscillations have been shown in both awake animals and in anesthetized animals, how these oscillations are associated with perception is beyond the scope of this presented research. To understand how the integration of brain state and the visual-evoked gamma oscillations affects perception, one must create a behavioral paradigm in which animals report their response to visual stimuli.

Another limitation is that all mice were induced with isoflurane and measured under the two doses of isoflurane before they were given propofol. Therefore, under the propofol delivery, there may be a slight mixing effect with isoflurane. This paradigm was chosen because in the acute setting, our induction and insertion of the jugular venous catheter is best done with isoflurane. This is because isoflurane has fast on – off kinetics and is much easier to titrate during the long, invasive surgeries of jugular cannulation and craniotomy. To induce and maintained an animal with only propofol, one would need to chronically insert the jugular catheter, allow the animal to recover, and then induce the animal with propofol for neurosurgery and beginning the experiment. Moreover, the animal may need a relatively high dose of propofol for induction, which may take a long time to wash out to maintain a steady state low dose for visual stimulation. We attempted to correct for the amount of isoflurane present within the brain of animals by starting propofol delivery 45 min before recording visual evoked potentials with isoflurane off. Previous results by Yanagisawa et al. (2008) and Friedman et al. (2010) show that the brain has at most trace levels of isoflurane after 15 min after isoflurane is turned off. Therefore, after 45 min of isoflurane off and propofol infusion, there should be virtually no isoflurane within the mouse brain. We also monitored propofol washout kinetics using the model that was fit to reflect the elimination time constant from the brain (Shortal et al., 2018). Thus, while there is a trace amount of propofol in the brain during the re-exposure to isoflurane, the albeit incomplete, recovery of responses first observed under isoflurane suggest that the order of drug administration is not likely to be a major contributor to our findings.

Finally, through employing high density, multichannel recording methods, including ECoG as we have used here, we can start asking questions about how visual evoked activity that initiates in V1 propagate to other visual areas. Answering such questions requires extensive analysis of the dynamically changing correlation structure of the spontaneous and evoked activity across electrodes. Moreover, concluding how anesthesia affect the propagation of such signals will require further parameterization of the visual stimulus characteristics as well as brain state with different anesthetics.

## Conclusion

Here, we show that even when the spontaneous activity of the brain shows similar spectral features, i.e., delta power or burst suppression, visual evoked activity is better correlated with the anesthetic drug rather than with the ensuing spectral state of the brain. Therefore, the canonical methods of defining the brain state with spontaneous spectral activity are not complete. Recently there has been a resurgence in efforts to define the state of the brain as induced by different anesthetic agents. One promising approach uses phase based functional connectivity measures to determine the flow of information from one brain region to the other (Lee et al., 2013; Kim et al., 2016). Another interesting set of methods include observing changes in the dynamics of the correlations in the signals from multiple electrodes (Solovey et al., 2015). Critical to the success of these methods is the understanding of how these models cope with sensory perturbations. Therefore, in addition to studying spontaneous activity under anesthesia and wakefulness, it will also be important to observe sensory evoked activity.

## Materials and Methods

### Animals

All experiments in this study were approved by Institutional Animal Care and Use Committee at the University of Pennsylvania and were conducted in accordance with the National Institutes of Health guidelines. All experiments were performed using adult (12–28 weeks old, 20–30 g) male and female C57BL/6 mice (Jackson Laboratories). Mice were housed under a reverse 12:12 h, light: dark cycle. Mice were provided with food and water *ad libitum*. A total of 11 mice were recorded from in this study. Inclusion criteria for mice included the following: (1) presence of spontaneous activity that was not characterized as burst suppression at lower drug concentrations (2) presence of VEPs at each dose of each anesthetic (as defined by the absolute value of the average LFP response exceeding five standard deviations of pre-stimulus data within 200 ms after stimulus presentation). With this inclusion criteria, we present data from 7 mice in this study.

### Surgery

All surgery was performed under aseptic conditions. Each animal was weighed (20–30 g) immediately prior to surgery to adequately dose propofol delivery. Prior to surgery, 2 mg/kg of dexamethasone was given subcutaneously (SQ) to reduce brain edema. Animals were induced with 2.5% isoflurane in oxygen (flow rate 500 ml/min), and maintained at 1.5% isoflurane for the remainder of the surgery. Core-body temperature was maintained at 37 ( ± 0.5)°C using a temperature controller with core-body temperature monitoring (TC- 1000 Temperature Controller, CWE, Incorporated, Ardmore, PA, United States). First, a jugular venous catheter was placed prior to neurosurgery to allow for Targeted Controlled Infusion (TCI) propofol infusion as described in Shortal et al. (2018). Animals were then secured into a Kopf stereotaxic frame. 0.25 ml of 2% lidocaine gel was applied to the scalp to provide a local nerve blockade during surgery. The scalp was then incised and retracted, permitting maximum exposure of the surface of the skull. The bone was cleaned and dried before a craniotomy was performed using a dental drill. One large craniotomy was drilled over the left hemisphere (+1 mm to -5 mm AP, +0.25 mm to +6 mm ML of bregma), and a small reference screw was secured in the right skull bone (+1 mm AP, +1 mm ML of bregma). A 64-electrode surface grid (Neuronexus: E64-500-20-60) was positioned over the dura to obtain ECoG signal. Mineral oil was applied on top of the ECoG grid every 20 min to preserve the health of the underlying dura and brain. Animals were scarified the same day immediately after the final visual recording session.

### Visual Stimulation

Visual stimuli consisted of a brief 10 ms flash of a bright green LED light (0.43 mW/cm^2^), placed 2 cm away from the mouse’s right eye. The flash covered 100% of the mouse’s visual field. Hundred flashes were given under each anesthetic dose step.

### Anesthetic Delivery Protocol

After the jugular catheter was placed and the craniotomy was completed, the doses and anesthetics were parametrically altered. The animal was first given two doses of isoflurane (Terrell Isoflurane, Novaplus): 1.2% (high dose isoflurane) and 0.6% (low dose isoflurane). Animals always received the higher isoflurane concentration before receiving the lower dose. The brain was allowed 15 min to equilibrate after the amount of isoflurane was changed (Friedman et al., 2010). After the two isoflurane doses, the TCI propofol (1000 mg per 100 ml Diprivan, Fresenius Kabi, United States) was administered using the model described in Shortal et al. (2018). The two target concentrations were 20 μg/g brain (low dose propofol), and 35 μg/g brain (high dose propofol). One out of seven of the mice was producing spontaneous movement at the 20 μg/g brain dose of propofol. Therefore, this mouse received 30 μg/g brain for low dose propofol, and 40 μg/g brain for high dose propofol to maintain slow waves and burst suppression activity, respectively. Due to the slow rate of excretion of propofol, the lower concentration of propofol was administered before the higher dose. Due to the fast rate of onset of propofol, the equilibration time between propofol changes was 8 min. Animals were not intubated, nor was an arterial catheter placed for pCO_2_ or blood pressure measurement.

In four of the seven animals, re-exposure doses of isoflurane were given in order to control for possible brain desiccation effects or impedance changes from keeping a large craniotomy open for a long period of time. In these animals, after achieving the 35 μg/g brain concentration of propofol, the propofol infusion was turned off and 1.2% isoflurane was administered for 1 h in order to allow propofol to wash out. Propofol washout was monitored using the same TCI model used for propofol delivery. This model estimates the amount of propofol remaining in the brain parenchyma. Following this wash out period, visual stimuli were again given under 1.2 and 0.6% re-exposure isoflurane.

### Electrophysiology and Preprocessing

In six mice, signals were amplified via a Neuralynx headstage (HS36), digitized through Cheetah 64 acquisition system (Neuralynx, ERP-27, Lynx-8), and collected at a rate of 3030.3 samples/s. In one mouse, signals were amplified via an Intan headstage (Intan, RHD2132), digitized through Omniplex acquisition system (Plexon, Omniplex), and collected at a rate of 30,000 samples/s.

LFP data collected with Neuralyx was filtered online using a proprietary FIR filter between 0.1 and 325 Hz. LFP data collected with Plexon was filtered offline using a custom-built FIR filter between 0.1 Hz and 325 Hz, with the MATLAB functions, *firls.m* and *filtfilt.m*. Offline, both data sets were decimated to 1000 samples/s, noise channels were manually removed and trials with excess motion artifact or saturated data was rejected before the mean was subtracted from the data. All data analysis was completed using custom built Matlab (Mathworks) code unless otherwise stated.

### Selection of Electrode Over Primary Visual Cortex (V1)

The latency of onset of the VEP was calculated for each electrode in the array as the time point at which if their post-stimulus average exceeds three standard deviations above the pre-stimulus baseline for three consecutive time points. The electrode which had the lowest latency of onset was denoted as V1. The amplitude of the VEP was calculated by determining the RMS of the first 350 ms of post-stimulus data. This was determined for both raw voltage signals and for voltage signals normalized to 500 ms of pre-stimulus data and expressed as a z-score.

$$RMS=\sqrt{\frac{1}{n}\left(x_{1}^{2}+x_{2}^{2}+...+x_{n}^{2}\right)}$$

Where x is the voltage in the post-stimulus average, and n is the time point in ms. Duration of the VEP was defined as the first time point in which the post-stimulus data returns to within two standard deviations of the pre-stimulus data for 20 consecutive time points.

### Quantification of Reliability in Time Domain

To asses reliability of the LFP evoked response to the visual stimulus in time domain, a similar method was used to that of Tiesinga et al. (2003) and Kumbhani et al. (2007). First, pairwise correlations of single trial evoked potentials were computed over the first 350 ms of post-stimulus activity for each dose of each anesthetic. These correlations were then averaged together to compute reliability. Correlation was computed using the Matlab *corr.m* function.

Reliability=Σi=1trialsΣj=1+itrials(LFPi−μi)(LFPj−μj)/σi*σj(trials2−trials)/2

Where *LFP_i_* is the evoked response during the *i-*th trial, μ*_i_* and σ*_i_* are the mean and standard deviation of the single trial response.

### Quantification of Suppression Ratio (SR)

To asses determine which epochs of the LFP were suppressed, both a frequency based metric and an amplitude based matric was applied to the data. First, spectrograms were calculated using multi-taper spectral analysis by applying the MATLAB function, *swTFspecAnalog.m*, written by Dr. Andrew Hudson. Spectral analysis was performed from 2 to 500 Hz with a set of 5 Slepian tapers, over a window size of 500 ms, with 80% overlap. The total power was then calculated for each window for frequencies between 2 and 100 Hz. The resultant total power time series of a burst suppression data set (high dose isoflurane or high dose propofol) was then subjected to k-means clustering to find 2 centroids- one that would correspond to bursts, and the other to suppressions. From this, the maximum threshold for classifying suppression based on total power was calculated for each mouse. Concurrently, RMS was also calculated over the LFP data in 500 ms windows with 80% overlap. A manual maximum RMS threshold by eye was selected for each mouse. Time windows were classified as suppression as long as the total power and RMS were below their respective thresholds. The suppression ratio (SR) was calculated by number of time windows with suppression divided by the total number of time windows.

### Spectral Analysis of Spontaneous LFP

One minute of unstimulated LFP was extracted from each mouse under each concentration of isoflurane and propofol. Re-exposure isoflurane baseline data was excluded since excess propofol may have remained within the brain given the propofol’s slow excretion rate. Power spectral density of each segment was calculated using multi-taper spectral analysis by applying the MATLAB function, *mtpsd.m*, written by Dr. Andrew Hudson. Spectral analysis was performed from 0.05 to 100 Hz with a set of 20 slepian tapers. All power spectra were normalized to total power. The average power spectra and 95% confidence intervals for each concentration of isoflurane and propofol was calculated using the ensuing normalized power spectra. Spectra in Figure 2B are show on a log-log scale.

### Wavelet Analysis

Power, phase, and frequency information was extracted by convolving single trial data with a set of Morlet wavelets (0.1–150 Hz, with a step-width 0.25 Hz and normalized amplitude), generated with using continuous wavelet transform, *contwt.m*, in MATLAB, written by Christopher Torrence and Gilbert Compo^1^ (Torrence and Compo, 1998). The ensuing power spectrograms of single trial data were averaged within each mouse, under each concentration of isoflurane and propofol. The average spectrograms were then normalized to 300 ms of baseline data. Spectrograms shown in Figure 6 are shown with frequency in log space.

### Inter-trial Phase Coherence (ITPC) Analysis

Inter-trial phase coherence (ITPC) was used to quantify the amount of phase synchrony between trials at each frequency. ITPC over V1 in each mouse under each concentration of anesthetics. First, angle vectors were extracted from the wavelet coefficients at each time point and each frequency by applying Euler’s formula and setting the single trial vector length to 1. ITPC was then calculated by taking the mean length of the angle vector across trials. ITPC at each timepoint and frequency of all mice were averaged separately under isoflurane and propofol. Figure 7 shows the difference in the average visual-evoked ITPC between mice under isoflurane and propofol.

### Statistical Analysis

Statistics presented in Figure 3 were presented using Krustal–Wallis–Mann *U*-test for group comparison and a Mann–Whitney *U*-test for concentration specific effects. *P*-values were Bonferroni corrected for multiple comparisons among four groups. Figure 7 were performed using Mann–Whitney *U*-tests. *P*-values were made more stringent using a Bonferroni correction for multiple comparisons among 900 time points.

## Ethics Statement

All experiments in this study were approved by Institutional Animal Care and Use Committee at the University of Pennsylvania and were conducted in accordance with the National Institutes of Health guidelines. All experiments were performed using adult (12–28 weeks old, 20–30 g) male and female C57BL/6 mice (Jackson Laboratories). Mice were housed under a reverse 12:12 h, light: dark cycle. Mice were provided with food and water *ad libitum*.

## Author Contributions

AA, CB, DC, MK, and AP: conceptualization. AA, BS, DC, and AP: data curation. AA, CB, and AP: formal analysis. DC, MK, and AP: funding acquisition. AA and AP: writing – original draft. AA, CB, BS, MK, DC, and AP: writing – review and editing.

## Conflict of Interest Statement

The authors declare that the research was conducted in the absence of any commercial or financial relationships that could be construed as a potential conflict of interest.

## Footnotes

1 http://paos.colorado.edu/research/wavelets/

## References

[B1] Akeju O. Song A. H. Hamilos A. E. Pavone K. J. Flores F. J. Brown E. N. (2016). Electroencephalogram signatures of ketamine anesthesia-induced unconsciousness. *Clin. Neurophysiol.* 127 2414–2422. 10.1016/j.clinph.2016.03.005 27178861 PMC4871620

[B2] Amzica F. (2009). Basic physiology of burst-suppression. *Epilepsia* 50(Suppl. 12), 38–39. 10.1111/j.1528-1167.2009.02345.x 19941521

[B3] Arena A. Lamanna J. Gemma M. Ripamonti M. Ravasio G. Zimarino V. (2017). Linear transformation of the encoding mechanism for light intensity underlies the paradoxical enhancement of cortical visual responses by sevoflurane. *J. Physiol.* 595 321–339. 10.1113/JP272215 27416731 PMC5199737

[B4] Aston-Jones G. Cohen J. D. (2005). An integrative theory of locus coeruleus-norepinephrine function: adaptive gain and optimal performance. *Annu. Rev. Neurosci.* 28 403–450. 10.1146/annurev.neuro.28.061604.135709 16022602

[B5] Bai D. Pennefather P. S. MacDonald J. F. Orser B. A. (1999). The general anesthetic propofol slows deactivation and desensitization of GABA A receptors. *J. Neurosci.* 19 10635–10646. 10.1523/jneurosci.19-24-10635.1999 10594047 PMC6784967

[B6] Banks M. I. Pearce R. A. (1999). Dual actions of volatile anesthetics on GABAAIPSCs: dissociation of blocking and prolonging effects. *Anesthesiology* 90 120–134. 10.1097/00000542-199901000-00018 9915321

[B7] Cardin J. A. (2016). Snapshots of the brain in action: local circuit operations through the lens of γ oscillations. *J. Neurosci.* 36 10496–10504. 10.1523/jneurosci.1021-16.2016 27733601 PMC5059425

[B8] Cardin J. A. Carlén M. Meletis K. Knoblich U. Zhang F. Deisseroth K. (2009). Driving fast-spiking cells induces gamma rhythm and controls sensory responses. *Nature* 459 663–667. 10.1038/nature08002 19396156 PMC3655711

[B9] Ching S. Cimenser A. Purdon P. L. Brown E. N. Kopell N. J. (2010). Thalamocortical model for a propofol-induced -rhythm associated with loss of consciousness. *Proc. Natl. Acad. Sci. U.S.A.* 107 22665–22670. 10.1073/pnas.1017069108 21149695 PMC3012501

[B10] Destexhe A. Contreras D. Steriade M. (1999). Spatiotemporal analysis of local field potentials and unit discharges in cat cerebral cortex during. *J. Neurosci.* 19 4595–4608. 10.1523/jneurosci.19-11-04595.1999 10341257 PMC6782626

[B11] Eckenhoff M. F. (2002). Multiple specific binding targets for inhaled anesthetics in the mammalian brain. *J. Pharmacol. Exp. Ther.* 300 172–179. 10.1124/jpet.300.1.172 11752113

[B12] Eckenhoff M. F. Eckenhoff R. G. (1998). Quantitative autoradiography of halothane binding in rat brain. *J. Pharmacol. Exp. Ther.* 285 371–376. 9536033

[B13] Eldar E. Cohen J. D. Niv Y. (2013). The effects of neural gain on attention and learning. *Nat. Neurosci.* 16 1146–1153. 10.1038/nn.3428 23770566 PMC3725201

[B14] Ermentrout G. B. Kleinfeld D. (2001). Traveling electrical waves in cortex: insights from phase dynamics and speculation on a computational role. *Neuron* 29 33–44. 10.1016/s0896-6273(01)00178-7 11182079

[B15] Ferron J.-F. Kroeger D. Chever O. Amzica F. (2009). Cortical Inhibition during burst Suppression Induced with isoflurane anesthesia. *J. Neurosci.* 29 9850–9860. 10.1523/JNEUROSCI.5176-08.2009 19657037 PMC6666595

[B16] Franks N. P. (2008). General anaesthesia: from molecular targets to neuronal pathways of sleep and arousal. *Nat. Rev. Neurosci.* 9 370–386. 10.1038/nrn2372 18425091

[B17] Friedman E. B. Sun Y. Moore J. T. Hung H. T. Meng Q. C. Perera P. (2010). A conserved behavioral state barrier impedes transitions between anesthetic-induced unconsciousness and wakefulness: evidence for neural inertia. *PLoS One* 5:e11903. 10.1371/journal.pone.0011903 20689589 PMC2912772

[B18] Fries P. Neuenschwander S. Engel A. K. Goebel R. Singer W. (2001). Rapid feature selective neuronal synchronization through correlated latency shifting. *Nat. Neurosci.* 4 194–200. 10.1038/84032 11175881

[B19] Friston K. Buzsáki G. (2016). The functional anatomy of time: what and when in the brain. *Trends Cogn. Sci.* 20 500–511. 10.1016/j.tics.2016.05.001 27261057

[B20] Fu Y. Tucciarone J. M. Espinosa J. S. Sheng N. Darcy D. P. Nicoll R. A. (2014). A cortical circuit for gain control by behavioral state. *Cell* 156 1139–1152. 10.1016/j.cell.2014.01.050 24630718 PMC4041382

[B21] Garcia P. S. Kolesky S. E. Jenkins A. (2010). General anesthetic actions on GABA A receptors. *Curr. Neuropharmacol.* 8 2–9. 10.2174/157015910790909502 20808541 PMC2866459

[B22] Gibbs F. Gibbs E. Lennox W. (1937). Effect on the electro-encephalogram of certain drugs which influence nervous activity. *Arch. Intern. Med* 60 154–166.

[B23] Gilzenrat M. S. Nieuwenhuis S. Jepma M. Cohen J. D. (2010). Pupil diameter tracks changes in control state predicted by the adaptive gain theory of locus coeruleus function. *Cogn. Affect. Behav. Neurosci.* 10 252–269. 10.3758/CABN.10.2.252 20498349 PMC3403821

[B24] Hales T. G. Lambert J. J. (1991). The actions of propofol on inhibitory amino acid receptors of bovine adrenomedullary chromaffin cells and rodent central neurones. *Br. J. Pharmacol.* 104 619–628. 10.1111/j.1476-5381.1991.tb12479.x 1665745 PMC1908220

[B25] Hall A. C. Lieb W. R. Franks N. P. (1994). Stereoselective and non-stereoselective actions of isoflurane on the GABAA receptor. *Br. J. Pharmacol.* 112 906–910. 10.1111/j.1476-5381.1994.tb13166.x 7921619 PMC1910207

[B26] Hubel D. H. Wiesel T. N. (1962). Receptive fields, binocular interaction and functional architecture in the cat’s visual cortex. *J. Physiol.* 160 106–154. 10.1113/jphysiol.1962.sp006837 14449617 PMC1359523

[B27] Imas O. A. Ropella K. M. Ward B. D. Wood J. D. Hudetz A. G. (2005). Volatile anesthetics enhance flash-induced gamma oscillations in rat visual cortex. *Anesthesiology* 102 937–947. 10.1097/00000542-200505000-00012 15851880

[B28] Jones M. V. Harrison N. L. (1993). Effects of volatile anesthetics on the kinetics of inhibitory postsynaptic currents in cultured rat hippocampal neurons. *J. Neurophysiol.* 70 1339–1349. 10.1152/jn.1993.70.4.1339 7506753

[B29] Jurd R. Arras M. Lambert S. Drexler B. Siegwart R. Crestani F. (2003). General anesthetic actions in vivo strongly attenuated by a point mutation in the GABA A receptor β3 subunit. *FASEB J.* 17 250–252. 10.1096/fj.02-0611fje 12475885

[B30] Kim M. Mashour G. A. Moraes S. Vanini G. Tarnal V. Janke E. (2016). Functional and topological conditions for explosive synchronization develop in human brain networks with the onset of anesthetic-induced unconsciousness. *Front. Comput. Neurosci.* 10:1. 10.3389/fncom.2016.00001 26834616 PMC4720783

[B31] Kopell N. Ermentrout G. B. Whittington M. A. Traub R. D. (2000). Gamma rhythms and beta rhythms have different synchronization properties. *Proc. Natl. Acad. Sci. U.S.A.* 97 1867–1872. 10.1073/pnas.97.4.1867 10677548 PMC26528

[B32] Krasowski M. D. Koltchine V. V. Rick C. E. Ye Q. Finn S. E. Harrison N. L. (1998). Propofol and other intravenous anesthetics have sites of action on the gamma-aminobutyric acid type A receptor distinct from that for isoflurane. *Mol. Pharmacol.* 53 530–538. 10.1124/mol.53.3.530 9495821

[B33] Kroeger D. Amzica F. (2007). Hypersensitivity of the anesthesia-induced comatose brain. *J. Neurosci.* 27 10597–10607. 10.1523/jneurosci.3440-07.2007 17898231 PMC6673173

[B34] Kumbhani R. D. Nolt M. J. Palmer L. A. (2007). Precision, reliability, and information-theoretic analysis of visual thalamocortical neurons. *J. Neurophysiol.* 98 2647–2663. 10.1152/jn.00900.2006 17581854

[B35] Lee A. M. Hoy J. L. Bonci A. Wilbrecht L. Stryker M. P. Niell C. M. (2014). Identification of a brainstem circuit regulating visual cortical state in parallel with locomotion. *Neuron* 83 455–466. 10.1016/j.neuron.2014.06.031 25033185 PMC4151326

[B36] Lee U. Ku S. Noh G. Baek S. Choi B. Mashour G. A. (2013). Disruption of frontal–parietal communication by ketamine, propofol, and sevofluran. *Anesthesiology* 118 1264–1275. 10.1097/ALN.0b013e31829103f5 23695090 PMC4346246

[B37] Leslie K. Chan M. T. V. Myles P. S. Forbes A. McCulloch T. J. (2010). Posttraumatic stress disorder in aware patients from the B-aware trial. *Anesth. Analg.* 110 823–828. 10.1213/ANE.0b013e3181b8b6ca 19861364

[B38] Lydic R. Baghdoyan Helen A. (2005). Sleep, anesthesiology, and the neurobiology of arousal state control. *Anesthesiology* 103 1268–1295. 10.1097/00000542-200512010-00024 16306742

[B39] Maksimow A. Särkelä M. Långsjö J. W. Salmi E. Kaisti K. K. Yli-Hankala A. (2006). Increase in high frequency EEG activity explains the poor performance of EEG spectral entropy monitor during S-ketamine anesthesia. *Clin. Neurophysiol.* 117 1660–1668. 10.1016/j.clinph.2006.05.011 16807101

[B40] Mashour G. A. (2006). Integrating the science of consciousness and anesthesia. *Anesth. Analg.* 10 975–982. 10.1213/01.ane.0000232442.69757.4a 17000815

[B41] Mashour G. A. Orser B. A. Avidan M. S. (2011). Intraoperative awareness: from neurobiology to clinical practice. *Anesthesiology* 114 1218–1233. 10.1097/ALN.0b013e31820fc9b6 21464699

[B42] Mashour G. A. Shanks A. Tremper K. K. Kheterpal S. Turner C. R. Ramachandran S. K. (2012). Prevention of intraoperative awareness with explicit recall in an unselected surgical population. *Anesthesiology* 117 717–725. 10.1097/aln.0b013e31826904a6 22990178 PMC3447261

[B43] Miu P. Puil E. (1989). Isoflurane-induced impairment of synaptic transmission in hippocampal neurons. *Exp. Brain Res.* 75 354–360. 2542074 10.1007/BF00247941

[B44] Mountcastle V. B. (1957). Modality and topographic properties neurons of cat’s somatic sensory cortex. *J. Physiol.* 20 408–434. 10.1152/jn.1957.20.4.408 13439410

[B45] Niell C. M. Stryker M. P. (2010). Modulation of visual responses by behavioral state in mouse visual cortex. *Neuron* 65 472–479. 10.1016/j.neuron.2010.01.033 20188652 PMC3184003

[B46] Noda T. Takahashi H. (2015). Anesthetic effects of isoflurane on the tonotopic map and neuronal population activity in the rat auditory cortex. *Eur. J. Neurosci.* 42 2298–2311. 10.1111/ejn.13007 26118739

[B47] Pearce R. A. (1996). Volatile anaesthetic enhancement of paired-pulse depression investigated in the rat hippocampus in vitro. *J. Physiol.* 492(Pt 3), 823–840. 10.1113/jphysiol.1996.sp021349 8734993 PMC1158903

[B48] Pinto L. Goard M. J. Estandian D. Xu M. Kwan A. C. Lee S. H. (2013). Fast modulation of visual perception by basal forebrain cholinergic neurons. *Nat. Neurosci.* 16 1857–1863. 10.1038/nn.3552 24162654 PMC4201942

[B49] Polack P.-O. O. Friedman J. Golshani P. (2013). Cellular mechanisms of brain state-dependent gain modulation in visual cortex. *Nat. Neurosci.* 16 1331–1339. 10.1038/nn.3464 23872595 PMC3786578

[B50] Purdon P. L. Pierce E. T. Mukamel E. A. Prerau M. J. Walsh J. L. Foon K. (2013). Electroencephalogram signatures of loss and recovery of consciousness from propofol. *Proc. Natl. Acad. Sci. U.S.A.* 110 E1142–E1151. 10.1073/pnas.1221180110 23487781 PMC3607036

[B51] Ranft A. Kurz J. Deuringer M. Haseneder R. Dodt H. U. Zieglgänsberger W. (2004). Isoflurane modulates glutamatergic and GABAergic neurotransmission in the amygdala. *Eur. J. Neurosci.* 20 1276–1280. 10.1111/j.1460-9568.2004.03603.x 15341599

[B52] Reinhold K. Lien A. D. Scanziani M. (2015). Distinct recurrent versus afferent dynamics in cortical visual processing. *Nat. Neurosci.* 18 1789–1797. 10.1038/nn.4153 26502263

[B53] Russell I. F. (1989). Conscious awareness during general anaesthesia: relevance of autonomic signs and isolated arm movements as guides to depth of anaesthesia. *Baillieres. Clin. Anaesthesiol.* 3 511–532. 10.1016/s0950-3501(89)80016-9

[B54] Sanders R. (2016). Incidence of connected consciousness after tracheal intubation. *Anesthesiology* 126 214–222. 10.1097/ALN.0000000000001479 27984262

[B55] Sanders R. D. Tononi G. Laureys S. Sleigh J. W. (2012). Unresponsiveness ≠ unconsciousness. *Anesthesiology* 116 946–959. 10.1097/ALN.0b013e318249d0a7 22314293 PMC3311716

[B56] Schneider Gerhard M. D. Hollweck Regina M. S. Ningler Michael M. S. Stockmanns Gudrun P. D. Kochs Eberhard F. (2005). Detection of consciousness by electroencephalogram and auditory evoked potentials. *Anesthesiology* 103 934–943. 10.1097/00000542-200511000-00006 16249666

[B57] Sellers K. K. Bennett D. V. Hutt A. Fröhlich F. (2013). Anesthesia differentially modulates spontaneous network dynamics by cortical area and layer. *J. Neurophysiol.* 110 2739–2751. 10.1152/jn.00404.2013 24047911

[B58] Sellers K. K. Bennett D. V. Hutt A. Williams J. H. Fröhlich F. (2015). Awake vs. anesthetized: layer-specific sensory processing in visual cortex and functional connectivity between cortical areas. *J. Neurophysiol.* 113 3798–3815. 10.1152/jn.00923.2014 25833839 PMC4473519

[B59] Shortal B. P. Reitz S. L. Aggarwal A. Meng Q. C. McKinstry-Wu A. R. Kelz M. B. (2018). Development and validation of brain target controlled infusion of propofol in mice. *PLoS One* 13:e0194949. 10.1371/journal.pone.0194949 29684039 PMC5912730

[B60] Sohal V. S. (2016). How close are we to understanding what (if anything) γ oscillations do in cortical circuits? *J. Neurosci.* 36 10489–10495. 10.1523/jneurosci.0990-16.2016 27733600 PMC5059424

[B61] Solovey G. Alonso L. M. Yanagawa T. Fujii N. Magnasco M. O. Cecchi G. A. (2015). Loss of consciousness is associated with stabilization of cortical activity. *J. Neurosci.* 35 10866–10877. 10.1523/JNEUROSCI.4895-14.2015 26224868 PMC4518057

[B62] Sonner J. M. Werner D. F. Elsen F. P. Xing Y. Liao M. Harris R. A. (2007). Effect of isoflurane and other potent inhaled anesthetics on minimum alveolar concentration, learning, and the righting reflex in mice engineered to express α1 γ-aminobutyric acid type A receptors unresponsive to isoflurane. *Anesthesiology* 106 107–113. 10.1097/00000542-200701000-00019 17197852

[B63] Storchi R. Bedford R. A. Martial F. P. Allen A. E. Wynne J. Montemurro M. A. (2017). Modulation of fast narrowband oscillations in the mouse retina and dLGN according to background light intensity. *Neuron* 93 299–307. 10.1016/j.neuron.2016.12.027 28103478

[B64] Tang P. Eckenhoff R. (2018). Recent progress on the molecular pharmacology of propofol. *F1000Res.* 7:123. 10.12688/f1000research.12502.1 29445451 PMC5791003

[B65] Tiesinga P. Whitmer D. Sejnowski T. J. Fellous J. M. Schreiber S. (2003). A new correlation-based measure of spike timing reliability. *Neurocomputing* 5 925–931. 10.1016/s0925-2312(02)00838-x 20740049 PMC2926980

[B66] Torrence C. Compo G. P. (1998). A practical guide to wavelet analysis. *Bull. Am. Meteorol. Soc.* 79 61–78.

[B67] Traub R. D. Contreras D. Cunningham M. O. Murray H. Fiona E. N. Roopun A. (2016). Single-column thalamocortical network model exhibiting gamma oscillations, sleep spindles, and epileptogenic bursts single-column thalamocortical network model exhibiting gamma oscillations, sleep spindles, and epileptogenic bursts. *J. Neurophysiol.* 93 2194–2232. 10.1152/jn.00983.2004 15525801

[B68] Traub R. D. Whittington M. A. Stanford I. M. Jefferys J. G. R. (1996). A mechanism for generation of long-range synchronous fast oscillations in the cortex. *Nature* 383 621–624. 10.1038/383621a0 8857537

[B69] Vinck M. Batista-Brito R. Knoblich U. Cardin J. A. (2015). Arousal and locomotion make distinct contributions to cortical activity patterns and visual encoding. *Neuron* 86 740–754. 10.1016/j.neuron.2015.03.028 25892300 PMC4425590

[B70] Wang X. (2009). Propofol and isoflurane enhancement of tonic gamma-aminobutyric acid type a current in cardiac vagal neurons in the nucleus ambiguus. *Anesth. Analg.* 108 142–148. 10.1213/ane.0b013e31818d8b79 19095842

[B71] Wang X. Y. Buzsáki G. (1996). Gamma oscillation by synaptic inhibition in a hippocampal interneuronal network model. *J. Neurosci.* 16 6402–6413. 10.1523/jneurosci.16-20-06402.1996 8815919 PMC6578902

[B72] Weiser B. P. Hall M. A. Weinbren N. L. Woll K. A. Dailey W. P. Eckenhoff M. F. (2015). Macroscopic and macromolecular specificity of alkylphenol anesthetics for neuronal substrates. *Sci. Rep.* 5 1–6. 10.1038/srep09695 25853337 PMC4894431

[B73] Welle C. G. Contreras D. (2015). Sensory-driven and spontaneous gamma oscillations engage distinct cortical circuitry. *J. Neurophysiol.* 115 1821–1835. 10.1152/jn.00137.2015 26719085 PMC4869477

[B74] Whittington M. A. Traub R. D. Kopell N. Ermentrout B. Buhl E. H. (2000). Inhibition-based rhythms: Experimental and mathematical observations on network dynamics. *Int. J. Psychophysiol.* 38 315–336. 10.1016/s0167-8760(00)00173-2 11102670

[B75] Wilson H. R. Cowan J. D. (1972). Excitatory and inhibitory interactions in localized populations of model neurons. *Biophys. J.* 12 1–24. 10.1016/s0006-3495(72)86068-5 4332108 PMC1484078

[B76] Womelsdorf T. Fries P. Mitra P. P. Desimone R. (2006). Gamma-band synchronization in visual cortex predicts speed of change detection. *Nature* 439 733–736. 10.1038/nature04258 16372022

[B77] Womelsdorf T. Lima B. Vinck M. Oostenveld R. Singer W. Neuenschwander S. (2012). Orientation selectivity and noise correlation in awake monkey area V1 are modulated by the gamma cycle. *Proc. Natl. Acad. Sci. U.S.A.* 109 4302–4307. 10.1073/pnas.1114223109 22371570 PMC3306673

[B78] Yanagisawa M. Sun Y. Kelz M. B. Thornton M. Moore J. T. Chen J. (2008). An essential role for orexins in emergence from general anesthesia. *Proc. Natl. Acad. Sci. U.S.*A. 105 1309–1314. 10.1073/pnas.0707146105 18195361 PMC2234134

[B79] Yip G. M. S. Chen Z. W. Edge C. J. Smith E. H. Dickinson R. Hohenester E. (2013). A propofol binding site on mammalian GABA A receptors identified by photolabeling. *Nat. Chem. Biol.* 9 715–720. 10.1038/nchembio.1340 24056400 PMC3951778

